# Enhanced heterogenous hydration of SO_2_ through immobilization of pyridinic-N on carbon materials

**DOI:** 10.1098/rsos.192248

**Published:** 2020-08-19

**Authors:** Longhua Zou, Ping Yan, Peng Lu, Dongyao Chen, Wei Chu, Wanglai Cen

**Affiliations:** 1 Institute of New Energy and Low-carbon Technology, Sichuan University, Chengdu 610065, People's Republic of China; 2 College of Architecture and Environment, Sichuan University, Chengdu 610065, People's Republic of China; 3 National Engineering Research Center for Flue Gas Desulfurization, Sichuan University, Chengdu 610065, People's Republic of China; 4 The Key Laboratory of Water and Air Pollution Control of Guangdong Province, South China Institute of Environmental Sciences, Ministry of Ecology and Environment of China, Guangzhou 510655, People's Republic of China

**Keywords:** graphene, nitrogen doping, desulfurization, density functional theory, hydration

## Abstract

Carbon materials doped with nitrogen have long been used for SO_2_ removal from flue gases for the benefits of the environment. The role of water is generally regarded as hydration of SO_3_ which is formed through the oxidization of SO_2_. However, the hydration of SO_2_, especially on the surface of N-doped carbon materials, was almost ignored. In this study, the hydration of SO_2_ was investigated in detail on the pyridinic nitrogen (PyN)-doped graphene (GP) surfaces. It is found that, compared with the homogeneous hydration of SO_2_ assisted with NH_3_ in gas phase, the heterogeneous hydration is much more thermodynamically and kinetically favourable. Specifically, when a single H_2_O molecule is involved, the energy barrier for SO_2_ hydration is as low as 0.15 eV, with 0.59 eV released, indicating the hydration of SO_2_ can occur at rather low water concentration and temperature. Thermodynamic integration molecular dynamics results show the feasibility of the hydrogenated substrate recovery and the immobilized N acting as a catalytic site for SO_2_ hydration. Our findings show that the heterogeneous hydration of SO_2_ should be universal and potentially uncover the puzzling reaction mechanism for SO_2_ catalytic oxidation at low temperature by N-doped carbon materials.

## 1. Introduction

The use of carbon-based materials for sulfur dioxide (SO_2_) removal can be dated back to the 1980s [1–5]. It features the reaction of SO_2_ in the presence of O_2_ and H_2_O which involves a series of reactions to produce sulfuric acid as the final product at relatively low temperature (20–150°C) conditions. It is conventionally recognized that SO_2_ is oxidized by O* coming from the dissociation of adsorbed O_2_ molecule to form SO_3_ [6,7]. While the role of water is generally regarded as the hydration of SO_3_ to form sulfuric acid and flush it out from the micropores of carbon materials [3,8]. The mechanism ignored the effects and participation of H_2_O in the early stages for SO_2_ adsorption and its oxidation. However, since the concentration of water vapour in the flue gas is usually as high as 10 vol%, the hydration of adsorbed SO_2_ on the carbon surface, especially N-doped carbon materials, is feasible. Furthermore, our previous published work [9] following the mechanism shows that an optimized energy barrier for SO_2_ catalytic oxidation is in the range of 0.5–0.6 eV, which is still too high to explain the high performance of carbon materials for SO_2_ removal at low temperature. There should be a new mechanism to address these issues, where the H_2_O effects should be taken into account in the early stages.

Substantial efforts have been dedicated to investigate the hydration of SO_2_ in aqueous aerosols or gas phase both from experimental [10] and computational perspectives [11,12]. The adsorption and subsequent hydration process lead to the wet deposition of SO_2_, which has a significant impact on the formation of sulfate aerosols [13]. It is noteworthy that ‘sulfurous acid’ (H_2_SO_3_) has never been characterized or isolated experimentally due to its short lifetime. It can be easily split into SO_2_ and H_2_O [14]. Theoretical studies reveal that the gaseous hydration reaction of SO_2_ without assistant is unfavourable. While the addition of water, NH_3_ [15] or sulfuric acid [16] exhibits evident promotion effects by reducing the reaction barrier, due to a proton transmitter effect. Especially, the basic NH_3_ molecule can promote the gaseous hydration of SO_2_ both thermodynamically and kinetically, where SO_2_ accepts OH^−^ to form ${\text{HSO}}_{3}^{\;-}$ and NH_3_ accepts H^+^ to form NH_4_^+^. Recently, Lv *et al*. [17] reported that methylamine (MA) and dimethylamine (DMA) can also enhance SO_2_ hydration with even lower energy barriers than ammonia. With only two H_2_O molecules involved, the DMA-assisted SO_2_ hydration process is nearly barrierless. Inspired by the promotion effects of basic molecules for SO_2_ hydration in gas phase or gas/water interface, we assume that the immobilization of basic pyridinic N groups on carbon materials can make the SO_2_ hydration occur on the water–carbon interface in a heterogeneous way.

Actually, the incorporation of basic functional groups into carbon materials has been proved as an effective way to increase the adsorption and conversion of SO_2_ [18,19]. Among them, the non-metallic nitrogen groups are most widely investigated for their high electron-donating capacity [20,21]. Therefore, the charge density and distribution of the original graphene (GP) lattice are modulated, inducing the formation of local activation regions [22,23] to enhance the interaction between carbon materials and polar molecules [24]. The nitrogen-containing groups mainly consist of pyridinic, pyrrolic and graphitic configurations. Among them, the pyridinic N (PyN), a N atom bonded with two C atoms and as a member of hexagon in GP lattice, has been experimentally confirmed the most basic one for introduction of strong electron donor states near the Fermi level [25,26]. And Raymundo-Piñero *et al*. [27] demonstrated that performance enhancement for SO_2_ catalytic conversion is primarily related to the doping of PyN. Moreover, the doping of PyN is thermodynamically preferable [9], which usually occurs at the boundary of vacancy sites or at the edge of layers [28]. Through modulation of the C/N ratio of the precursors [25] and type of catalysts [29] during the preparation process, the pyridinic N is the predominant doping type to be obtained. Recently, a three PyN atoms-doped mono-vacancy structure has been synthesized and observed directly by scanning tunnelling microscopy (STM) [30], which makes it possible for precise tuning of PyN doping in the lattice of carbon-based catalysts.

Herein, we focused on the heterogeneous hydration of SO_2_ on a range of PyN-doped GP substrates to uncover (i) the feasibility for the promotion of SO_2_ hydration in thermodynamics and kinetics, (ii) the local structure of the active centre, and (iii) the hydration products and their implications for the total SO_2_ catalytic oxidation. It is interesting to find that the hydration of SO_2_ on PyN atoms-doped GP is much more preferable than the process in gaseous phase, indicating the important role of the water–carbon interface and a new reaction pathway for SO_2_ catalytic oxidation.

## 2. Calculation details

**Figure 1.**
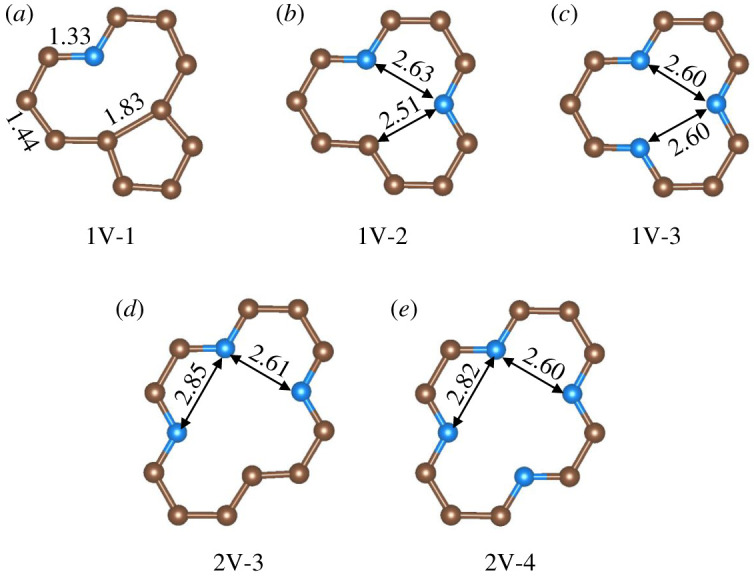
Local structures of relaxed pyridinic N-doped graphene models. Blue and brown spheres depict N and C atoms, respectively. Selected bond lengths are given in Å.

A 3√3 × 6 supercell of GP consisting of 72 C atoms was used as the basic model, with relaxed lattice parameters of 12.78 × 14.76 Å^2^. A vacuum region of 15 Å was added perpendicular to the plane to avoid interaction between neighbouring layers. Mono- and di-vacancy were created in the plane of GP by removing one or two adjacent C atoms. Experimental [31] and our previous theoretical study [9] revealed that the unsaturated C atoms on the vacancy site of GP are inclined to be substituted by N atoms, namely substitution in the form of pyridinic N (PyN). When *x* (*x* = 1–3) PyN atoms are introduced to the boundary of the mono-vacancy substrate, it is denoted as 1V-*x*. Following the same rule, the three and four PyN atoms doped di-vacancy substrate are denoted as 2V-3 and 2V-4, respectively (figure 1).

All the spin-polarized density functional theory (DFT) calculations were carried out using the Perdew–Burke–Ernzerhof (PBE) [32] functional with DFT-D3 correction [33] as implemented in the Vienna ab initio simulation package (VASP 5.4) [34,35]. A plane-wave basis set with energy cut-off of 400 eV was employed within the framework of the projector-augmented wave (PAW) method [36]. The Brillouin zone was sampled using a Monkhorst–Pack 3 × 3 × 1 k-points mesh. Gaussian smearing with a smearing width of 0.2 eV was used. All the atoms were allowed to relax until the maximum Hellman–Feynman force on each atom was less than 0.02 eV Å^−1^, except the atoms on the boundary which were fixed in all directions. All the parameters used have been validated with reasonable accuracy in our previous works [6,37,38]. Climbing image nudged elastic band (CI-NEB) [39,40] method was employed to trace the minimum energy pathways (MEP) from an initial state (IS) to its final state (FS). The transition state (TS) was confirmed with a single imaginary frequency. The zero-point energy (ZPE) corrections were included for all energetic analysis. The adsorption energy Δ*E*_ads_ is defined as

$$\Delta E_{\mathrm{ads}\;}=\;E_{\mathrm{tot}}-(E_{\mathrm{mol}}\;+\;E_{\mathrm{sub}}),$$

where the *E*_tot_, *E*_mol_ and *E*_sub_ are the total energies of the adsorption complex, isolated molecule and PyN-doped GP substrate, respectively.

For thermodynamic integration [41] calculations, the reaction trajectory was firstly explored by the metadynamic [42] method. Then, 10 ps constrained molecular dynamics simulation was carried out for each selected image. Canonical ensemble (with constant particles number N, volume V, and temperature T) at 300 K was used with timestep of 1 fs. The mass of H atom was set to 2 arb. units. Refer to electronic supplementary material for detailed parameters and description.

## 3. Results and discussion

### 3.1. Adsorption of SO_2_ and H_2_O

**Figure 2.**
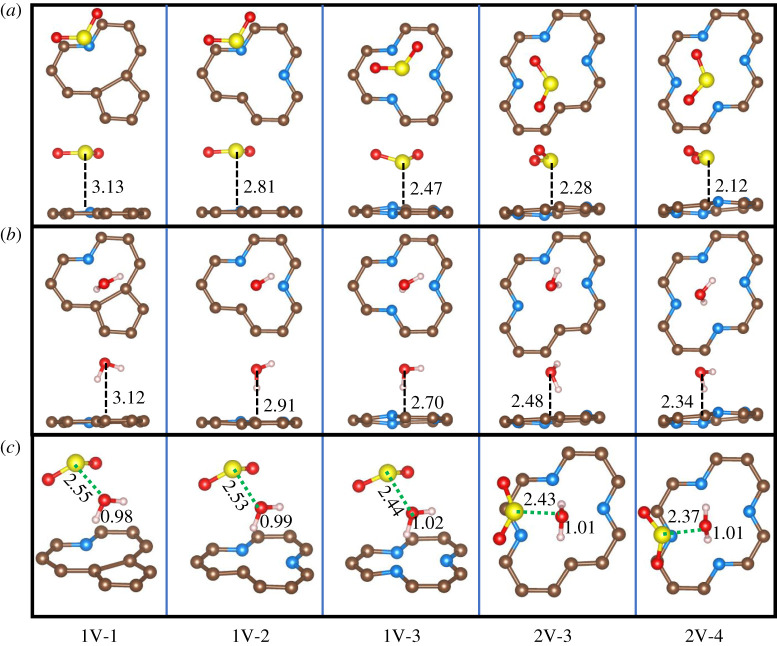
Adsorption configurations of SO_2_ (*a*), H_2_O (*b*) and their co-adsorption (*c*) on different PyN-doped structures. The yellow, red and light pink balls depict S, O and H atoms, respectively. The others are depicted in the same way as figure 1. The distances are given in Å.

**Figure 3.**
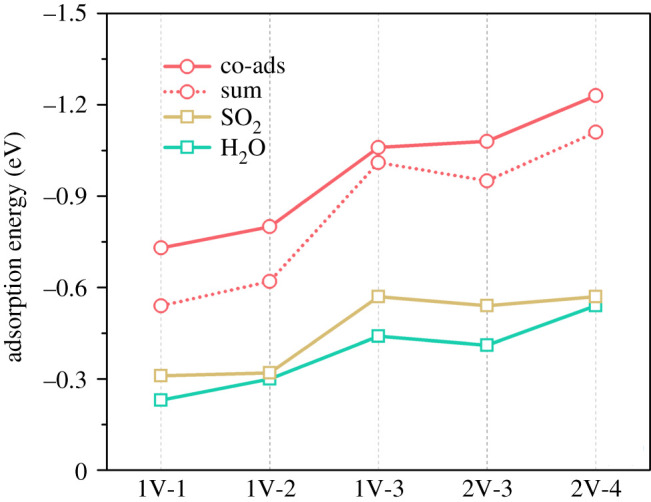
Adsorption energy of H_2_O and SO_2_ on different PyN-doped structures. The ‘sum’ is simply the summation of the adsorption energy for separate H_2_O and SO_2_ molecules.

As shown in figure 2*a*, when a single PyN atom is introduced (1V-1), the SO_2_ molecule is inclined to be parallel to the base plane, with the S atom atop of the PyN atom with a height of 3.13 Å. When a second PyN atom is added (1V-2), the adsorption configuration almost remains the same but with a shorter height of 2.81 Å. If more PyN atoms are introduced, as shown in 1V-3, 2V-3 and 2V-4, the adsorbed SO_2_ molecule is drawn closer to the centre of the vacancy with the S atom slightly tilted down. The variation of the adsorption configurations and the monotonic increase in the adsorption energy (figure 3) with the increase of PyN atoms indicate that the adsorption of SO_2_ can be enhanced by PyN doping. It should be due to the Lewis basicity of PyN sites and the more PyN sites, the stronger the basicity [43]. For the adsorption of H_2_O (figure 2*b*), in each of the configurations, the H_2_O molecule is adsorbed with one H atom pointing down to the vacancy. Following the same trend for SO_2_ adsorption, the adsorption distance decreases according to the increase of PyN atoms number. It implies that the vacancy acts as a centre with negative charges, which is subject to adsorbing H_2_O and SO_2_ through static Coulomb interaction. However, the adsorption energy for H_2_O is lower than that for SO_2_. It might be due to more positive charge of S atom in SO_2_ than H in H_2_O.

For the co-adsorption of H_2_O and SO_2_, considering the concentration of H_2_O (approx. 10 vol%) in real flue gases is higher than that of SO_2_ (approx. 0.1 vol%) with an order of magnitude about 100, one H_2_O molecule was pre-absorbed before the introduction of SO_2_. The adsorption configurations and energies are shown in figures 2*c* and 3, respectively. Geometrically, the adsorption distance from the S atom of SO_2_ to the O atom of H_2_O is gradually shortened from 2.55 to 2.37 Å. Additionally, the bond length of the H–O pointing to the basal plane is elongated from 0.98 to 1.01–1.02 Å. They denote an increased interaction between SO_2_ and H_2_O molecules, which may favour the hydration reaction of SO_2_ by H_2_O. The enhanced interaction can also be confirmed by the co-adsorption energy, which is higher than the sum of the separate adsorption energy of SO_2_ and H_2_O. This trend is consistent with experimental studies [44] that the nitrogen atoms in carbon materials behave as the main polar sites, which is beneficial for adsorption of polar molecules such as H_2_O and SO_2_.

### 3.2. Products of SO_2_ hydration

**Figure 4.**
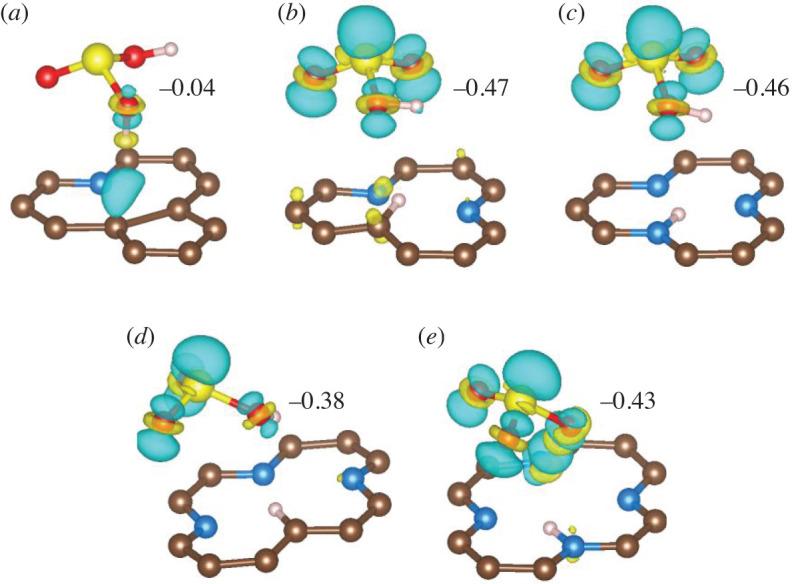
Charge difference and Bader population analysis of the products derived from SO_2_ hydration on different PyN-doped structures. The isosurface is set to 0.1 eV Å^−3^. The blue areas denote electron accumulation and yellow ones denote depletion.

Figure 4 shows the structures and charge states of SO_2_ hydration products based on several possible configurations tested (electronic supplementary material, figure S1). On 1V-1, the most possible product is H_2_SO_3_. For the other four situations, the thermodynamically feasible product is ${\text{HSO}}_{3}^{\;\delta -}$. Based on Bader population analysis, the value of *δ* is approximately 0.4–0.5. Considering the uncertainty of the Bader method and the geometry of ${\text{HSO}}_{3}^{\;\delta -}$, the product should be attributed to bisulfite. If more H_2_O molecules are included, which is practical in reaction conditions, they can help to stabilize ${\text{HSO}}_{3}^{\;\delta -}$ and the *δ* might be much closer to 1. Actually, when 7 and 24 H_2_O molecules are included, the calculated *δ* are 0.71 and 0.83, respectively (electronic supplementary material, figure S2 and table S1).

**Figure 5.**
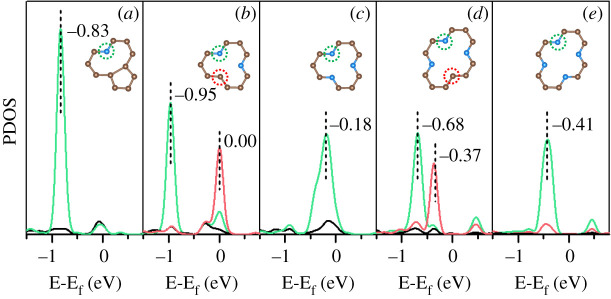
The highest occupied projected density of states (PDOS) for different PyN-doped substrates. (*a*) 1V-1, (*b*) 1V-2, (*c*) 1V-3, (*d*) 2V-3 and (*e*) 2V-4. The energy of Fermi level (E_f_) was reset to 0 eV, and the value of E-E_f_ refers to a relative energy to Fermi level. Corresponding N or C atoms are circled with dashed line in the same colour. States of an irrelated C atom far from the vacancy is also presented (black line) as reference.

When ${\text{HSO}}_{3}^{\;\delta -}$ is formed, the H atom dissociated from H_2_O molecule can preferably be accepted by the unsaturated C atom (e.g. 1V-2 and 2V-3) of the PyN-doped substrates, then by one of the N atoms (e.g. 1V-3 and 2V-4). This kind of selectivity can be addressed that, for 1V-3 (figure 5*c*) and 2V-4 (figure 5*e*), the PyN atoms are the centres of negative charge and the highest occupied states of the substrate were localized on them, which is an object for H ion adsorption. For 1V-2 (figure 5*b*) and 2V-3 (figure 5*d*), the highest occupied states of the unsaturated C atom are on top of N atoms and are much closer to the Fermi level, resulting in the C site being more preferable than N for H acceptance.

### 3.3. Reaction pathway of SO_2_ hydration

**Figure 6.**
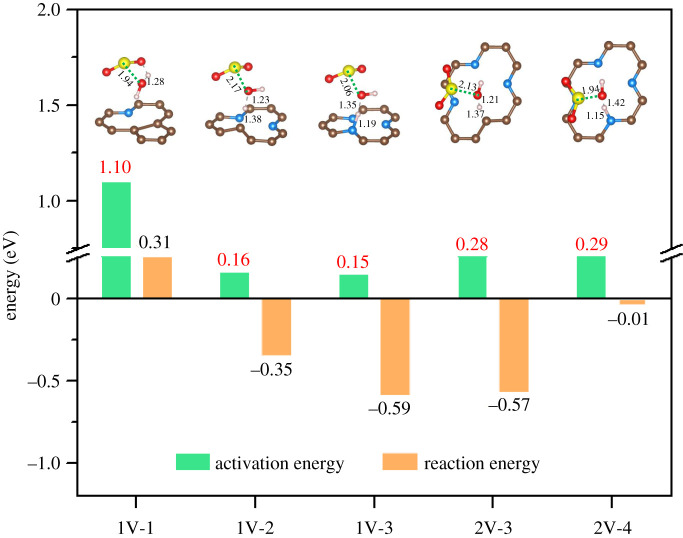
Reaction barriers (green bar) and energies (yellow bar) for SO_2_ hydration on different PyN-doped substrates. Local structures of the transition states (TS) are included as insets.

Figure 6 shows the energetics for the hydration reaction of SO_2_ on different PyN-doped configurations corresponding to the products shown in figure 5. The hydration of SO_2_ with single H_2_O molecule on 1V-1 is not preferable, with 0.31 eV cost. The corresponding barrier is as high as 1.10 eV, which is comparable to the value for SO_2_ hydration in gas phase [15,45,46]. The kinetically unpreferable hydration can be explained by the local structure of its transition states (TS) since a four-membered ring S–O–H–O with high inner tension was formed [15].

For the other four substrates, it is interesting to note that, all the hydration processes are thermodynamically preferable, with a certain energy released. Additionally, all the barriers for SO_2_ hydration decrease to a value below 0.30 eV. Specifically, the energy barrier is 0.15 eV on 1V-3, which is lower than that assisted with ammonia [15] (0.54 eV, transferred from 12.53 kcal mol^−1^) and MA (0.32 eV, transferred from 7.41 kcal mol^−1^) and DMA [17] (0.21 eV, transferred from 4.78 kcal mol^−1^) in the gas phase. The much lower barriers should be due to the hydration process following a different reaction pathway, where the H_2_O molecule is dissociating with one H atom approaching to the C or N atom of the substrate, meanwhile, the remaining OH reaching the S atom of SO_2_ to form bisulfite as the product. In summary, when the number of doped PyN atoms is more than one, the reaction of hydration of SO_2_ is feasible, both thermodynamically and kinetically.

Geometrically, the four feasible TS configurations can be categorized into two classes: the earlier TS class including 1V-2 and 2V-3, where the O–H bond is on the way to be broken and the H atom will be accepted by the unsaturated C atom, with H–C distance of 1.38 and 1.37 Å; and the latter TS class including 1V-3 and 2V-4, where the O–H bond has been completely broken and the H atom has been accepted by N atom, with a shorter H–N distance of 1.19 and 1.15 Å. The electronic local function (ELF, electronic supplementary material, figure S3) results denote stronger covalent interaction of H–N (0.93 for 2V-3 and 0.95 for 2V-4) than that of H–C (0.89 for 1V-2 and 0.90 for 2V-3).

However, the H atom acceptant C or N does not explain the inconsistent hydration barrier. For 2V-3 and 2V-4, they have different H acceptant, but share almost the same hydration barrier (0.28 and 0.29 eV). The same situation is found for 1V-2/3, but with much lower hydration barrier (0.16 and 0.15 eV). It should be partially addressed by the stronger co-adsorption of SO_2_ and H_2_O on 2V-3/4 than on 1V-2/3 for the larger vacancy in size of the former, according to the adsorption energy results shown in figure 3.

**Figure 7.**
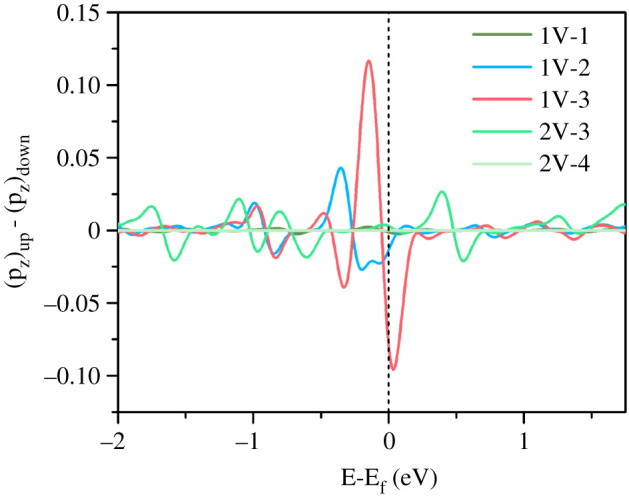
Spin-polarized 2p_z_ of the highest occupied states of different PyN-doped substrates: N for 1V-1, 1V-3, 2V-4 and C for 1V-2 and 2V-3. The Fermi level is set to 0 eV.

Furthermore, it should be noted the barrier for SO_2_ hydration increases in the order 1V-3 < 1V-2 < 2V-3 < 2V-4 < 1V-1. Comparing with the centre position of the highest occupied states in PDOSs 1V-2 > 1V-3 > 2V-3 > 2V-4 > 1V-1 (figure 5), the two series qualitatively match well except for 1V-2 and 1V-3. Correspondingly, figure 7 shows the spin-polarized p_z_ states of all the highest occupied states. It was calculated directly by subtracting the spin-down states from the spin-up states. A higher spin-polarized p_z_ state should be more feasible to accept H ion through covalent interaction, which can help to reduce the barrier of SO_2_ hydration through H_2_O dissociation. There are three intensive signals just below the Fermi level in the order 1V-3 > 1V-2 > 2V-3, indicating the lowest SO_2_ hydration barrier on 1V-3 and remedying the increase order 1V-3 < 1V-2.

Hence, we infer that the doping of negatively charged PyN atoms and the spin polarization states of C or N atoms cooperatively control the activity of PyN-doped substrates for SO_2_ hydration. Compared with 2V-3/4, the electrons around the vacancy of 1V-2/3 are extruded and split, leading to much higher occupied C/N 2p states at the energy level and extra unpaired electrons of C 2p_z_ and N 2p_z_ perpendicular to the plane of the substrates. Therefore, the introduction of basic PyN groups can modulate the in-plane local electronic structure and enhances its electrostatic force to adsorb polar H_2_O and SO_2_ molecules, while the asymmetric spin density out of plane dominates the chemical interaction and acts as selective sites for GP functionalization with proton coming from the dissociation of H_2_O. The two factors result in the most active centre with three PyN atoms-doped mono-vacancy structure 1V-3 for SO_2_ hydration.

### 3.4. Effects of extra water molecules

Since H_2_O is one of the majorities in fossil fuel burned flue gases and its presence is essential for SO_2_ catalytic oxidation by nitrogen-doped carbon materials at low temperature, the role of H_2_O should not be limited to an absorbent of the product SO_3_ as proposed conventionally. Here, we found the SO_2_ can be hydrated feasibly to form bisulfite, which might be readily oxidized in further steps to form sulfuric acid. The oxidation pathway has been suggested recently based on experiment results on water microdroplets [47,48]. To generalize our findings, the effects of H_2_O for SO_2_ hydration should be discussed in two points: (i) what are the effects if more H_2_O molecules present for SO_2_ hydration on PyN-doped substrate, and (ii) can the hydrogenated substrates be dehydrogenated to recycle as a catalyst?

Firstly, we propose that the hydration will be enhanced, specifically by reducing the barrier and increasing the total energy released to a limit. This trend has been reported for gaseous hydration reaction of SO_2_ assisted by NH_3_ and its derivatives of MA and DMA as mentioned before. For NH_3_, the barrier for *n* = 2 is about 0.25 eV (transferred from 5.9 kcal mol^−1^ in [13] and it is consistent with our calculated value *ca* 0.24 eV as shown in electronic supplementary material, figure S4) and it begins to release energy till *n* = 3. It is the same situation for amine, only when the number of water molecules is equal to or more than two, the hydration can be exothermic. For more H_2_O molecules involved in the reaction, the inner tension of the TS structure can be reduced to promote the hydration process. However, even when a single H_2_O molecule is included, the barrier is 0.15 eV on 1V-3, with 0.59 eV released. On 2V-4, although the reaction energy is irrelevant −0.01 eV, the value will increase to −0.06 and −0.31 eV when extra one and six H_2_O molecules are added, respectively (electronic supplementary material, figure S5). This is probably because the asymmetric spin density out of plane can significantly reduce the ring structure tension, thus the TS is more stable. On the other hand, this kind of enhancement effects both on barrier decrease and reaction energy increase can be attributed to the nature of the interface between PyN-doped carbon surface and the adsorbed H_2_O cluster. From the perspective of electronic structure, the introduction of pyridinic N atoms into the GP lattice changes the in-plane charge distribution around the dopant site. This leads to weak electrostatic forces, which promote the adsorption of polar molecules H_2_O. A water cluster is inclined to be adsorbed on the PyN-doped site for its electronegativity and polarity through hydrogen bonding interaction, then facilitate the dissociation of H_2_O for SO_2_ hydration as NH_3_ and amine does in gaseous phase.

**Figure 8.**
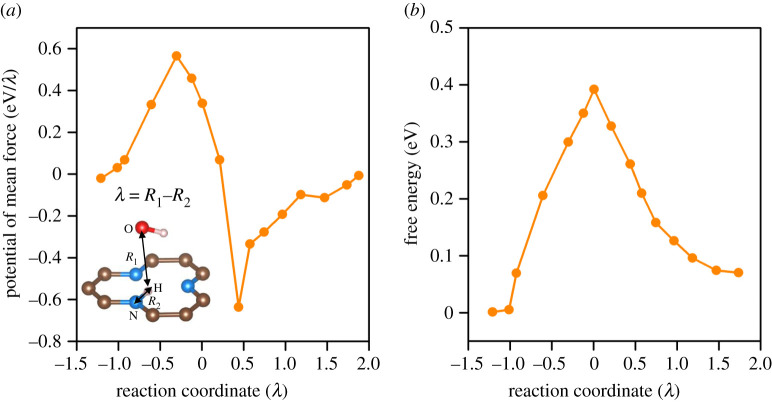
Potential of mean force (PMF) (*a*) and free energy profiles (*b*) along the reaction coordinate for dissociation of H ion from the 1V-3 substrate. The collective variable is chosen as *λ* = *R*_1_ − *R*_2_, where *R*_1_ and *R*_2_ is the distance of H–O and N–H as shown in the inset of (*a*).

In order to investigate the dehydrogenation feasibility of the substrate after SO_2_ hydration reaction, the free energy profile of the dehydrogenation of the hydrogenated pyridinic N was investigated with thermodynamic integration methods (refer to electronic supplementary material for details). Firstly, water dissociation was observed during the metadynamics simulation with Gaussian hills applied (electronic supplementary material, figure S6). Subsequently, thermodynamic integration calculations show that the H_2_O molecule dissociation process is endothermic (figure 8), indicating the reverse process for recovery of the hydrogenated substrate is thermodynamically preferable. This kind of dehydrogenation should be controlled by the influence of entropy [47], with a free energy decrease *ca* 0.1 eV and free energy barrier *ca* 0.3 eV, which are both kinetically and thermodynamically preferable. The simulation results indicate that the protonated substrate can be dehydrogenated and recovered for the next cycle of reaction.

## 4. Conclusion

Based on density functional theory calculations, it was found that SO_2_ can be hydrated readily on the surface of pyridinic N (PyN)-doped carbon materials. Compared with the process in the gaseous phase, the gas–solid interface enhances the hydration of SO_2_, both thermodynamically and kinetically. The hydrated product can be attributed to bisulfite (${\text{HSO}}_{3}^{\;-}$). Both the unsaturated C atoms or PyN atoms can act as active sites to accept H atoms to be hydrogenated, depending on their energy level of the highest occupied states and spin polarization. The most active sites should be the three PyN-doped mono-vacancy, with an energy barrier as low as 0.15 eV. When more H_2_O molecules are included, the heterogenous hydration of SO_2_ can be further facilitated, and the hydrogenated sites can be recovered for the next hydration reaction. Our work shed insights on the important role of the water–carbon interface for SO_2_ hydration and potentially opens a new reaction pathway for SO_2_ catalytic oxidation.

## Supplementary Material

Supporting Information.docx

Reviewer comments

## Supplementary Material

ESM - CAPTIONS.docx

## Supplementary Material

ESM-FIG1.pdf

## Supplementary Material

ESM-FIG2.pdf

## Supplementary Material

ESM-FIG3.pdf

## Supplementary Material

ESM-FIG4.pdf

## Supplementary Material

ESM-FIG5.pdf

## Supplementary Material

ESM-FIG6.pdf

## Supplementary Material

ESM - TABLE.docx

## Data accessibility

Our data are available from the Dryad Digital Repository: https://dx.doi.org/10.5061/dryad.6hdr7sqwr [49].

## References

[1] Lizzio AA, DeBarr JA 1997 Mechanism of SO_2_ removal by carbon. Energy Fuels 11, 284–291. (10.1021/ef960197+)

[2] Yan Z, Liu L, Zhang Y, Liang J, Wang J, Zhang Z, Wang X 2013 Activated semi-coke in SO_2_ removal from flue gas: selection of activation methodology and desulfurization mechanism study. Energy Fuels 27, 3080–3089. (10.1021/ef400351a)

[3] Raymundo-Piñero E, Cazorla-Amorós D, Salinas-Martinez de Lecea C, Linares-Solano A. 2000 Factors controling the SO_2_ removal by porous carbons: relevance of the SO_2_ oxidation step. Carbon 38, 335–344. (10.1016/S0008-6223(99)00109-8)

[4] Gaur V, Asthana R, Verma N 2006 Removal of SO_2_ by activated carbon fibers in the presence of O_2_ and H_2_O. Carbon 44, 46–60. (10.1016/j.carbon.2005.07.012)

[5] Kaneko K, Nakahigashi Y, Nagata K 1988 Microporosity and adsorption characteristics against NO, SO_2_, and NH_3_ of pitch-based activated carbon fibers. Carbon 26, 327–332. (10.1016/0008-6223(88)90223-0)

[6] Hou M, Zhang X, Yuan S, Cen W 2019 Double graphic-N doping for enhanced catalytic oxidation activity of carbocatalysts. Phys. Chem. Chem. Phys. 21, 5481–5488. (10.1039/C8CP07317A) 30783640

[7] He G, Ma J, He H 2018 Role of carbonaceous aerosols in catalyzing sulfate formation. ACS Catal. 8, 3825–3832. (10.1021/acscatal.7b04195)

[8] Sun X, Wang R, Su D 2013 Research progress in metal-free carbon-based catalysts. Chin. J. Catal. 34, 508–523. (10.1016/S1872-2067(11)60515-9)

[9] Li J, Liu J, Yin S, Liu Y, Li J, Cen W, Chu Y 2016 Promotion mechanism of pyridine N-doped carbocatalyst for SO_2_ oxidation. RSC Adv. 6, 86 316–86 323. (10.1039/C6RA17349G)

[10] Dermota TE, Hydutsky DP, Bianco NJ, Castleman AW 2005 Ultrafast dynamics of the SO_2_(H_2_O)n Cluster system. J. Phys. Chem. A 109, 8254–8258. (10.1021/jp052531l) 16834212

[11] Shamay ES, Johnson KE, Richmond GL 2011 Dancing on water: the choreography of sulfur dioxide adsorption to aqueous surfaces. J. Phys. Chem. C 115, 25 304–25 314. (10.1021/jp2064326)

[12] Donaldson DJ, Guest JA, Goh MC 1995 Evidence for adsorbed SO_2_ at the aqueous-air interface. J. Phys. Chem. 99, 9313–9315. (10.1021/j100023a002)

[13] Zhong J, Zhu C, Li L, Richmond GL, Francisco JS, Zeng XC 2017 Interaction of SO_2_ with the surface of a water nanodroplet. J. Am. Chem. Soc. 139, 17 168–17 174. (10.1021/jacs.7b09900) 29083178

[14] Townsend TM, Allanic A, Noonan C, Sodeau JR 2012 Characterization of sulfurous acid, sulfite, and bisulfite aerosol systems. J. Phys. Chem. A 116, 4035–4046. (10.1021/jp212120h) 22471624

[15] Liu J, Fang S, Liu W, Wang M, Tao F-M, Liu J-y 2015 Mechanism of the gaseous hydrolysis reaction of SO_2_: effects of NH_3_ versus H_2_O. J. Phys. Chem. A 119, 102–111. (10.1021/jp5086075) 25495573

[16] Liu J, Fang S, Wang Z, Yi W, Tao F-M, Liu J-y 2015 Hydrolysis of sulfur dioxide in small clusters of sulfuric acid: mechanistic and kinetic study. Environ. Sci. Technol. 49, 13 112–13 120. (10.1021/acs.est.5b02977) 26450714

[17] Lv G, Nadykto AB, Sun X, Zhang C, Xu Y 2018 Towards understanding the role of amines in the SO_2_ hydration and the contribution of the hydrated product to new particle formation in the Earth's atmosphere. Chemosphere 205, 275–285. (10.1016/j.chemosphere.2018.04.117) 29702347

[18] Menéndez JA, Radovic LR 1996 Low-temperature generation of basic carbon surfaces by hydrogen spillover. J. Phys. Chem. 100, 17 243–17 248.

[19] Mangun CL, DeBarr JA, Economy J 2001 Adsorption of sulfur dioxide on ammonia-treated activated carbon fibers. Carbon 39, 1689–1696. (10.1016/S0008-6223(00)00300-6)

[20] Paraknowitsch JP, Thomas A 2013 Doping carbons beyond nitrogen: an overview of advanced heteroatom doped carbons with boron, sulphur and phosphorus for energy applications. Energy Environ. Sci. 6, 2839–2855. (10.1039/C3EE41444B)

[21] Wang H, Maiyalagan T, Wang X 2012 Review on recent progress in nitrogen-doped graphene: synthesis, characterization, and its potential applications. ACS Catal. 2, 781–794. (10.1021/cs200652y)

[22] Zhang L, Xia Z 2011 Mechanisms of oxygen reduction reaction on nitrogen-doped graphene for fuel cells. J. Phys. Chem. C 115, 11 170–11 176. (10.1021/jp201991j)

[23] Groves MN, Chan ASW, Malardier-Jugroot C, Jugroot M 2009 Improving platinum catalyst binding energy to graphene through nitrogen doping. Chem. Phys. Lett. 481, 214–219. (10.1016/j.cplett.2009.09.074)

[24] Shen W, Fan W 2012 Nitrogen-containing porous carbons: synthesis and application. J. Mater. Chem. A 1, 999–1013. 10.1039/c2ta00028h

[25] Bulusheva L, Okotrub A, Fedoseeva Y, Kurenya A, Asanov I, Vilkov O, Koós A, Grobert N 2015 Control of incorporation of pyridinic, pyrrolic, graphitic, and molecular nitrogen in multi-wall carbon nanotubes via the N/C ratio in aerosol assisted chemical vapor deposition. Phys. Chem. Chem. Phys. 17, 23 741–23 747. (10.1039/C5CP01981H) 26104737

[26] Qu Z, Sun F, Liu X, Gao J, Zhipeng Q, Zhao G 2018 The effect of nitrogen-containing functional groups on SO_2_ adsorption on carbon surface: enhanced physical adsorption interactions. Surf. Sci. 677, 78–82. (10.1016/j.susc.2018.05.019)

[27] Raymundo-Piñero E, Cazorla-Amorós D, Linares-Solano A 2003 The role of different nitrogen functional groups on the removal of SO_2_ from flue gases by N-doped activated carbon powders and fibres. Carbon 41, 1925–1932. (10.1016/S0008-6223(03)00180-5)

[28] Pels JR, Kapteijn F, Moulijn JA, Zhu Q, Thomas KM 1995 Evolution of nitrogen functionalities in carbonaceous materials during pyrolysis. Carbon 33, 1641–1653. (10.1016/0008-6223(95)00154-6)

[29] Qu L, Liu Y, Baek J.-B., Dai L 2010 Nitrogen-doped graphene as efficient metal-free electrocatalyst for oxygen reduction in fuel cells. ACS Nano 4, 1321–1326. (10.1021/nn901850u) 20155972

[30] Ma C, Liao Q, Sun H, Lei S, Zheng Y, Yin R, Zhao A, Li Q, Wang B 2018 Tuning the doping types in graphene sheets by N monoelement. Nano Lett. 18, 386–394. (10.1021/acs.nanolett.7b04249) 29266951

[31] Gao Y, Hu G, Zhong J, Shi Z, Zhu Y, Su DS, Wang J, Bao X, Ma D 2013 Nitrogen-doped sp^2^-hybridized carbon as a superior catalyst for selective oxidation. Angew. Chem. Int. Ed. 52, 2109–2113. (10.1002/anie.201207918) 23307693

[32] Perdew JP, Burke K, Ernzerhof M 1996 Generalized gradient approximation made simple. Phys. Rev. Lett. 77, 3865–3868. (10.1103/PhysRevLett.77.3865) 10062328

[33] Grimme S 2010 Semiempirical GGA-type density functional constructed with a long-range dispersion correction. J. Comput. Chem. 27, 1787–1799. (10.1002/jcc.20495) 16955487

[34] Kresse G, Furthmüller J 1996 Efficient iterative schemes for ab initio total-energy calculations using a plane-wave basis set. Phys. Rev. B 54, 11 169–11 186. (10.1103/PhysRevB.54.11169) 9984901

[35] Kresse G, Furthmüller J 1996 Efficiency of ab-initio total energy calculations for metals and semiconductors using a plane-wave basis set. Comput. Mater. Sci. 6, 15–50. (10.1016/0927-0256(96)00008-0) 9984901

[36] Kresse G J., Joubert D 1999 From ultrasoft pseudopotentials to the projector augmented-wave method. Phys. Rev. B 59, 1758 (10.1103/PhysRevB.59.1758)

[37] Hou M, Cen W, Nan F, Li J, Chu Y, Yin H 2016 Dissociation of O_2_ and its reactivity on O/S doped graphene. RSC Adv. 6, 7015–7021. (10.1039/C5RA16789B)

[38] Li J, Shi Y, Dong F, Cen W, Chu Y 2017 Tailoring active sites via synergy between graphitic and pyridinic N for enhanced catalytic efficiency of a carbocatalyst. ACS Appl. Mater. Interfaces 9, 19 861–19 869. (10.1021/acsami.7b04026) 28534396

[39] Henkelman G 2000 A climbing image nudged elastic band method for finding saddle points and minimum energy paths. J. Chem. Phys. 113, 9901–9904. (10.1063/1.1329672)

[40] Henkelman G, Jónsson H 2000 Improved tangent estimate in the nudged elastic band method for finding minimum energy paths and saddle points. J. Chem. Phys. 113, 9978–9985. (10.1063/1.1323224)

[41] Fleurat-Lessard P, Ziegler T 2005 Tracing the minimum-energy path on the free-energy surface. J. Chem. Phys. 123, 084101 (10.1063/1.1948367) 16164276

[42] Laio A, Parrinello M 2002 Escaping free-energy minima. Proc. Natl Acad. Sci. USA 99, 12 562–12 566. (10.1073/pnas.202427399) PMC130499 12271136

[43] Mu X, Yuan B, Feng X, Qiu S, Lei S, Yuan H 2016 Effect of doped heteroatoms (nitrogen, boron, phosphorus) on inhibition thermal oxidation of reduced graphene oxide. RSC Adv. 6, 105 021–105 029. (10.1039/C6RA21329D)

[44] Lahaye J, Nansé G, Bagreev A, Strelko V 1999 Porous structure and surface chemistry of nitrogen containing carbons from polymers. Carbon 37, 585–590. (10.1016/S0008-6223(98)00225-5)

[45] Voegele AF, Tautermann CS, Rauch C, Loerting T, Liedl KR 2004 On the formation of the sulfonate ion from hydrated sulfur dioxide. J. Phys. Chem. A 108, 3859–3864. (10.1021/jp0377578)

[46] Sinha RK, Scuderi D, Maitre P, Chiavarino B, Crestoni ME, Fornarini S 2015 Elusive sulfurous acid: gas-phase basicity and IR signature of the protonated species. J. Phys. Chem. Lett. 6, 1605–1610. (10.1021/acs.jpclett.5b00450) 26263321

[47] Hung H-M, Hsu M-N, Hoffmann MR 2018 Quantification of SO_2_ oxidation on interfacial surfaces of acidic micro-droplets: implication for ambient sulfate formation. Environ. Sci. Technol. 52, 9079–9086. (10.1021/acs.est.8b01391) 30040406

[48] Hung H-M, Hoffmann MR 2015 Oxidation of gas-phase SO_2_ on the surfaces of acidic microdroplets: implications for sulfate and sulfate radical anion formation in the atmospheric liquid phase. Environ. Sci. Technol. 49, 13 768–13 776. (10.1021/acs.est.5b01658) 26270804

[49] Zou L, Yan P, Lu P, Chen D, Chu W, Cen W 2020 Data from: Enhanced heterogenous hydration of SO_2_ through immobilization of pyridinic-N on carbon materials. Dryad Digital Repository. (10.5061/dryad.6hdr7sqwr) PMC7481677 32968503

