# Supplementary material for: Enhanced heterogenous hydration of SO2 through immobilization of pyridinic-N on carbon materials
Source: R Soc Open Sci. 2020 Aug 19;7(8):192248. doi: 10.1098/rsos.192248 (PMC7481677; doi:10.1098/rsos.192248)
Supplement: ESM - CAPTIONS.docx [file rsos192248supp2.docx]

Supporting Information

**Enhanced Heterogenous Hydration of SO_2_ Through Immobilization of Pyridinic-N on Carbon Materials**

Longhua Zou^a^, Ping Yan^b^, Peng Lu^c^, Dongyao Chen^c^, Wei Chu^a^*, Wanglai Cen^a,d,^*

a. Insitute of New Energy and Low-carbon Technology, Sichuan University, Chengdu 610065, China

b. College of Architecture and Environment, Sichuan University, Chengdu 610065, China

c. The Key Laboratory of Water and Air Pollution Control of Guangdong Province, South China Institute of Environmental Sciences, Ministry of Ecology and Environment of China, Guangzhou 510655, China.

d. National Engineering Research Center for Flue Gas Desulfurization, Sichuan University, Chengdu, 610065, China.

**Corresponding Authors**

**Wei Chu** (Prof.): chuwei1965@scu.edu.cn

Add.: No.24 South Section 1, Yihuan Road, Chengdu, China, 610065

**Wanglai Cen** (Associate Prof.): cenwanglai@163.com

Add.: No.24 South Section 1, Yihuan Road, Chengdu, China, 610065

**Figure S1**. All the possible relaxed SO_2_ hydration product configurations on the five different PyN doped GPs and the most preferable ones are red squared on the first column. The total energies are labeled below in eV. Bond lengths are given in Å.

**Figure S2.** Relaxed geometry of HSO_3_ ^δ-^ with additional (a) 6 and (b) 23 H_2_O molecules.

**Table S1.** Bader population analysis of HSO_3_ ^δ-^ (O atoms are listed in Fig. S3) with different number of H_2_O molecules.

Note: ZVAL represent the number of valence electrons used in pseudopotentials files.

**Figure S3.** The electronic localized functions (ELFs) and line profiles for H (H_2_O) and C or N atom on GP substrate involved in the hydration process for the TS in 1V-2 (a), 1V-3 (b), 2V-3 (c) and 2V-4 (d).

**Figure S4.** NEB calculation for the reaction of SO_2_ + 2H_2_O + NH_3_ → NH_4_HSO_3_ + H_2_O.

**Figure S5.** The influence of additional (a) one, (b) six H_2_O molecules on the reaction energy of SO_2_ hydration on 2V-4. The effective charge based on Bader population analysis were labeled.

**Figure S6.** Evolution of the CV value (a) and the potential energy (b) during the metadynamics simulation for H_2_O dissociation process on 1V-3; (c) snapshots at 1, 1340 and 2500 fs, the bond breaking and formation process are highlighted. Brown, blue, red and pink balls represent C, N, O and H atoms respectively.

**Thermodynamic integration.** A supercell containing 1V-3 substrate and 24 H_2_O molecules was used as the model (14.76 x 12.78 x 25 Å). Before thermodynamic integration calculations, biased constrained molecular dynamics was run for 2.5 ps after 5 ps equilibration (BOMD method with NVT ensemble, T=300 K). The mass for H atom was set to 2 a.u. and the time step was set to 1 fs. A collective variable (CV) λ was defined as: λ = R_1_ − R_2_, where R_1_ is the distance of H-O of one selected H_2_O molecule and R_2_ is the distance of H-N. During the simulation process, the H-O bond is supposed to be broken and the H atom would be transferred to the N atom in the 1V-3 substrate. The height and width of the Gaussian hill were set to be 0.01 eV and 0.005 Å respectively for each 100 steps.

Based on the reaction trajectory found through the biased constrained molecular dynamics calculation, 14 windows were selected along the reaction path and thermodynamic integration calculations were run for 10 ps with NVT ensemble at 300K. The last 5000 steps were used for potential of mean force (PMF) calculation. The free energy barriers and free energy difference was obtained by trapezoidal integration of the PMF at each selected window.
