# Supplementary figures and images for: Enhanced heterogenous hydration of SO2 through immobilization of pyridinic-N on carbon materials
Source: R Soc Open Sci. 2020 Aug 19;7(8):192248. doi: 10.1098/rsos.192248 (PMC7481677; doi:10.1098/rsos.192248)

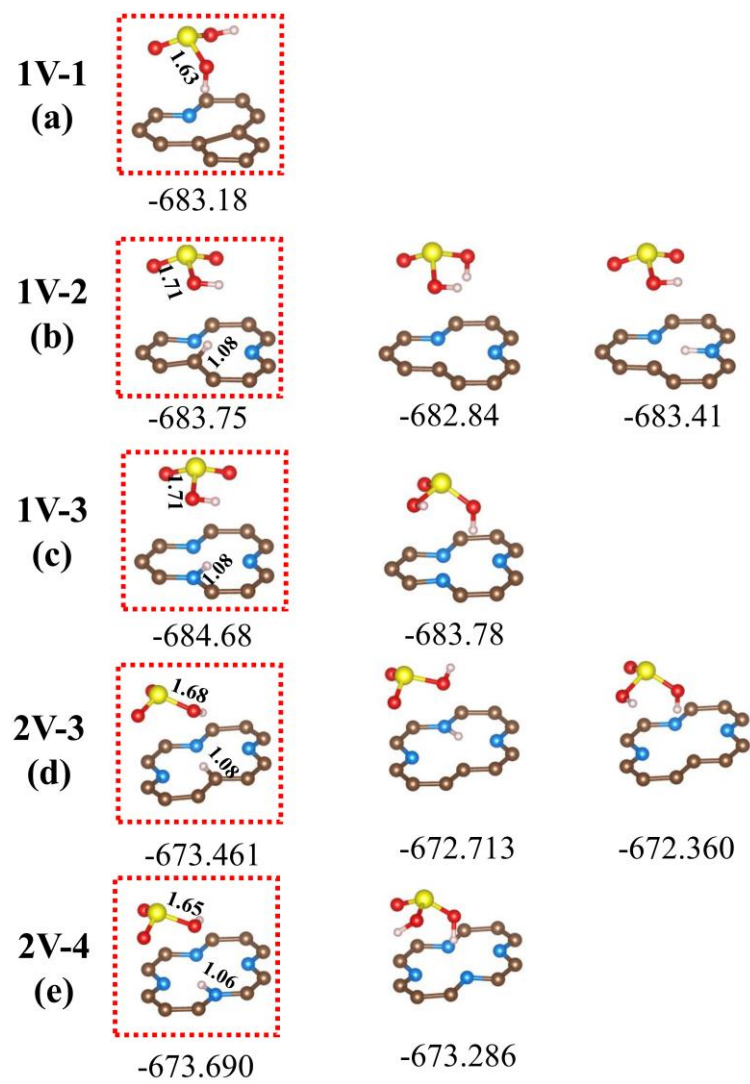

Supplement: ESM-FIG1.pdf [file rsos192248supp3.pdf]

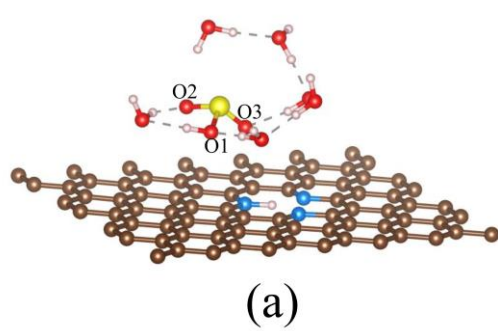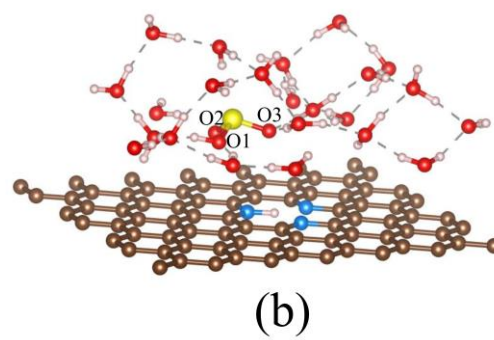

Supplement: ESM-FIG2.pdf [file rsos192248supp4.pdf]

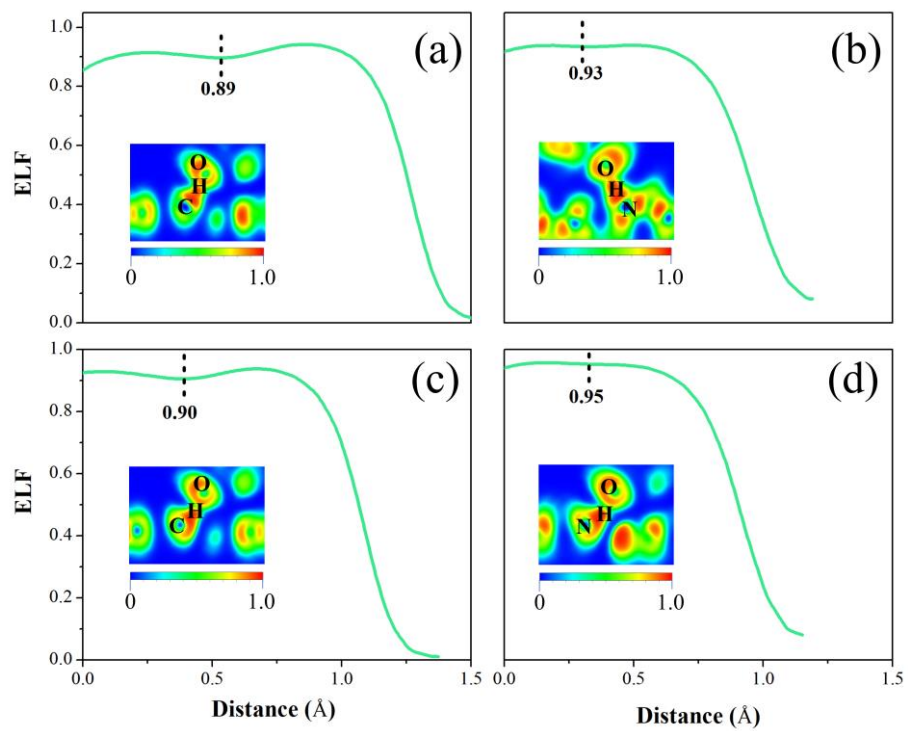

Supplement: ESM-FIG3.pdf [file rsos192248supp5.pdf]

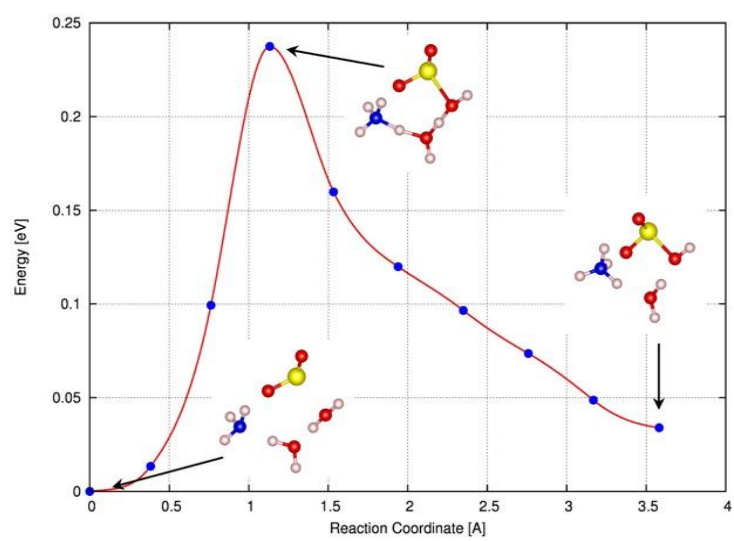

Supplement: ESM-FIG4.pdf [file rsos192248supp6.pdf]

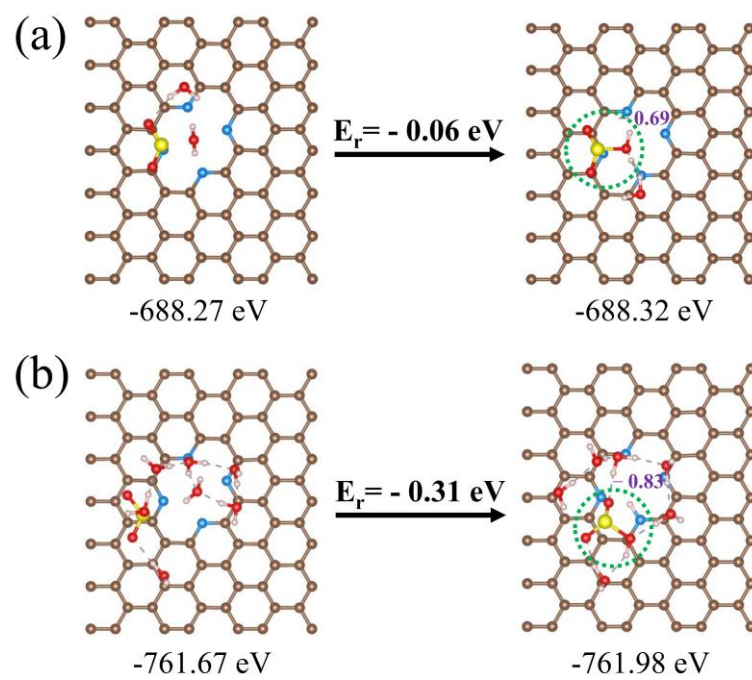

Supplement: ESM-FIG5.pdf [file rsos192248supp7.pdf]

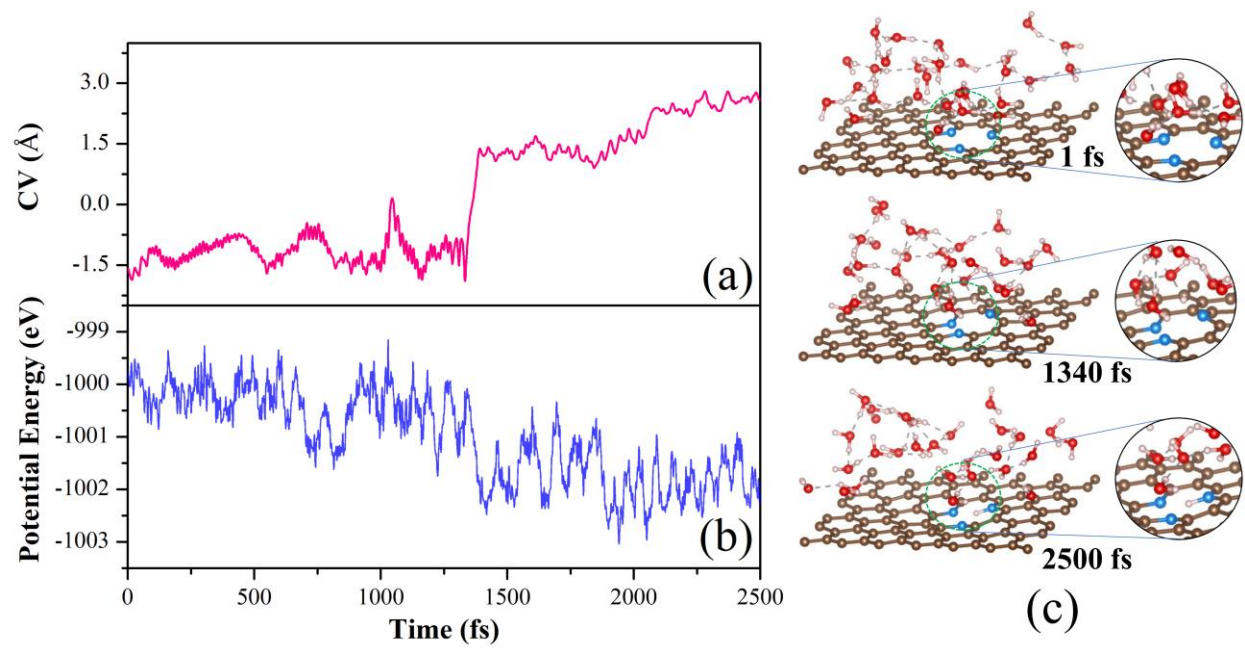

Supplement: ESM-FIG6.pdf [file rsos192248supp8.pdf]
