# Supplementary material for: Enhanced heterogenous hydration of SO2 through immobilization of pyridinic-N on carbon materials
Source: R Soc Open Sci. 2020 Aug 19;7(8):192248. doi: 10.1098/rsos.192248 (PMC7481677; doi:10.1098/rsos.192248)
Supplement: ESM - TABLE.docx [file rsos192248supp9.docx]

| **Items** | **O1** | **O2** | **O3** | **S** | **H** | **δ** |
| --- | --- | --- | --- | --- | --- | --- |
| 7H_2_O | 7.259 | 7.254 | 7.127 | 3.708 | 0.360 | 0.708 |
| 23H_2_O | 7.270 | 7.274 | 7.226 | 3.757 | 0.298 | 0.825 |
| ZVAL | 6 | 6 | 6 | 6 | 1 | / |

Note: ZVAL represent the number of valence electrons used in pseudopotentials files.
